# Neighbourhood characteristics, social capital and self-rated health - A population-based survey in Sweden

**DOI:** 10.1186/1471-2458-10-628

**Published:** 2010-10-21

**Authors:** Margareta Lindén-Boström, Carina Persson, Charli Eriksson

**Affiliations:** 1 Department of Community Medicine and Public Health, Örebro County Council, Sweden; 2 Department of Health Sciences, Örebro University, Sweden

## Abstract

**Background:**

In previous public health surveys large differences in health have been shown between citizens living in different neighbourhoods in the Örebro municipality, which has about 125000 inhabitants. The aim of this study was to investigate the determinants of health with an emphasis on the importance of neighbourhood characteristics such as the influence of neighbourhood social cohesion and social capital. The point of departure in this study was a conceptual model inspired by the work of Carpiano, where different factors related to the neighbourhood have been used to find associations to individual self-rated health.

**Methods:**

We used data from the survey 'Life & Health 2004' sent to inhabitants aged 18-84 years in Örebro municipality, Sweden. The respondents (n = 2346) answered a postal questionnaire about living conditions, housing conditions, health risk factors and individual health. The outcome variable was self-rated health. In the analysis we applied logistic regression modelling in various model steps following a conceptual model.

**Results:**

The results show that poor self-rated health was associated with social capital, such as lack of personal support and no experience of being made proud even after controlling for strong factors related to health, such as age, disability pension, ethnicity and economic stress. Also the neighbourhood factors, housing area and residential stability were associated with self-rated health. Poor self-rated health was more common among people living in areas with predominately large blocks of flats or areas outside the city centre. Moreover, people who had lived in the same area 1-5 years reported poor health more frequently than those who had lived there longer.

**Conclusions:**

The importance of the neighbourhood and social capital for individual health is confirmed in this study. The neighbourhoods could be emphasized as settings for health promotion. They can be constructed to promote social interaction which in turn supports the development of social networks, social support and social capital - all important determinants of health.

## Background

Health is unequally distributed in the population. Peoples' health varies, among other things, according to the neighbourhoods in which they live. The inhabitants of socio-economically weaker areas have higher morbidity as well as higher mortality [1,2]. The explanations for this can be both contextual and compositional. According to Blaxter, contextual effects are important. She writes, 'while the health of manual men and women was almost always poorer than that of non-manual, it is clear that types of living area do make a difference' [3]. Other researchers focus on compositional explanations and conclude that the concentration of people with adverse personal or household socio-economic characteristics in certain areas is the explanation for area variations in health [4-8]. A contextual explanation would be that there are some features of the neighbourhood that influence health. Contextual factors may either impact all residents in the same way or have a stronger impact on some of them (for instance women more than men). A compositional explanation for observed neighbourhood variations in health would be that people with poor health tend to live in certain areas and the place itself has no effect on their health. Differences in health between subjects in different neighbourhoods are attributable to the differences in the distribution of people with varying health status, not to other differences between neighbourhoods. In this study we are especially interested in studying contextual factors from an individual perspective.

Social capital is a notion which for the last two decades has been used to enhance the understanding of health and to explain differences in health. Bourdieu introduced the notion as 'the aggregate of the actual or potential resources which are linked to possession of a durable network of more or less institutionalized relationships of mutual acquaintance and recognition' [9]. However, Putnam is regarded as the researcher who made the concept more widely understood, particularly through his work from the early 1990s [10,11]. Trust is a vital component of social capital, according to Putnam. Trust facilitates cooperation, which in its turn leads to greater trust by strengthening mutual dependence among the individuals. Horizontal social networks in society - like for example athletic, cooperative, or neighbourhood associations - promote mutual norms and facilitate communication and the exchange of information [10,12,13]. Putnam has defined social capital as 'features of social organization, such as networks, norms and social trust, that facilitate coordination and cooperation for mutual benefit' [11]. In a later work he defines social capital as 'connections among individuals - social networks and the norms of reciprocity and trustworthiness that arise from them' [14]. Putnam's definition of trust is similar to the definition of social cohesion, a concept that Wilkinson used in his theory of the reasons for the unequal distribution of health [15]. It has been shown in several studies that different aspects of social capital covary with self-rated health [1,12,16-20]. Describing area-based differences in different health outcomes has a long history [2]. Clustering of individual health within neighbourhoods is far from being just a statistical nuisance; it is a key topic in social epidemiology [5,21-24]. However, statistical modelling separating individual from neighbourhood effects generally suggests a much larger effect on health of individual-level variation than neighbourhood-level variation [2]. A review of social capital and health revealed a significant health impact of both individual and area level social capital [16]. In one Swedish study, neighbourhood variance in self-rated health was mainly affected by factors other than individual social capital [25]. There are rival views regarding the concept social capital and it has been treated as a more sophisticated formulation of other concepts such as social cohesion [26], social support [12,27] and social integration [13].

There are large differences in health between different neighbourhoods in the Örebro municipality, which has about 125000 inhabitants. This was illustrated by dividing the municipality into areas with different neighbourhood characteristics such as city centre, residential areas, areas with blocks of flats and outlying areas. The largest proportion of poor self-rated health is found in areas with predominately blocks of flats. This is evident in the three population surveys from the years 1993, 2000 and 2004 [28-30]. In the 2004 survey 12% of the women aged 18-84 living in the areas with blocks of flats reported poor self-rated health compared to 5% in the city centre. The corresponding age-adjusted proportions among men were 15 and 5% respectively.

The purpose of this study is to investigate the determinants of health with an emphasis on the importance of the neighbourhood. We are especially interested in the importance of neighbourhood social cohesion and social capital. The outcome variable is self-rated health.

### Conceptual model

**Figure 1:**
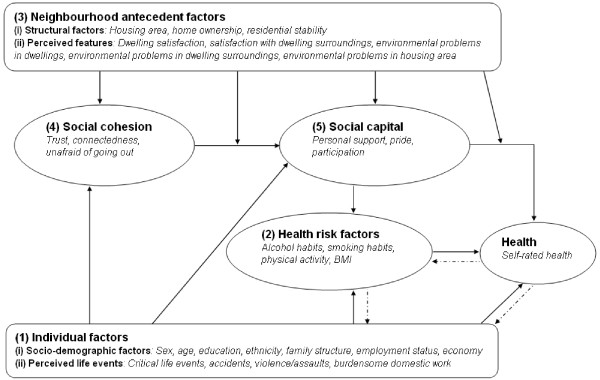
**Conceptual model of neighbourhood social capital processes on individual health**.

There is a considerable amount of research on social capital and health. To contribute to the theoretical structure of this research, Richard Carpiano has developed a conceptual model of neighbourhood social capital processes on individual health outcomes to explain the connection between neighbourhood antecedent factors, different components of social capital, individual characteristics and health [31,32]. It is a complex model, based on the theory of Bourdieu, and consists of both neighbourhood and individual levels. Founded on Carpiano's model, we were inspired to construct a modified model to use as a theoretical framework in the analysis of our data (Figure 1). A main difference between our model and Carpiano's is that we do not use a multilevel approach. The analysis is exclusively made at an individual-level.

#### Individual factors

Component 1, *individual factors*, consists of (i) socio-demographic factors and (ii) perceived life events. Socio-demographic factors are strongly related to health. Good health is more common among younger than older people, among natives than immigrants, and among married people than singles [33]. The individual social position constitutes a direct effect on health [34,35]. There are studies that show that the relative social position in the local social hierarchy has more influence on health than the absolute level of economic resources [36], but this has been debated [37]. Critical life events - often unwanted, uncontrollable and life-threatening situations - are included in perceived life events. Such life events have a negative influence on mental health [38] as well as on self-rated health [39]. A life 'event' of a different kind that has a strong relation to self-reported mental health is to experience domestic work at home as burdensome [40].

#### Health risk factors

Alcohol and smoking habits, physical activity, and body mass index (BMI) are included in component 2, *health risk factors*. High alcohol consumption constitutes a health hazard [41]. The connection between smoking and morbidity/mortality has been known for a long time [42]. There are strong relationships between health and physical activity and between health and body mass. There exists, furthermore, a relationship between physical inactivity, overweight and obesity.

#### Neighbourhood antecedent factors

Component 3 in the model, *neighbourhood antecedent factors*, consists of: (i) structural factors, like type of housing in the neighbourhood, home ownership, and residential stability as well as factors that are connected to the inhabitants´ experience of the neighbourhood, (ii) perceived features, such as satisfaction with the dwelling and its surroundings. Neighbourhood antecedent factors impact the living-conditions of residents and create and maintain social attachment and ultimately social capital. It has been shown that factors that are connected to the actual neighbourhood and the inhabitants' experience of it can partly explain the observed differences in health [43,44].

#### Social cohesion

*Social cohesion *is often included in the concept of social capital, but in line with Carpiano's model, it is a component of its own, number 4. In this model based on Bourdieu's thesis, social cohesion is regarded as a precondition that enables social capital to be developed and used in action [10]. Feelings of togetherness with other inhabitants and a sense of security in the neighbourhood are included in this concept. People who live in areas with low levels of trust between the inhabitants have a higher rate of poor self-rated health than those in other neighbourhoods [17].

#### Social capital

Component 5 in the model, *social capital*, depends mainly on the availability of social support, but also includes activity in organizations. To be involved in activities related to organizations is in fact to be included in social networks, which in turn strengthen one's personal resources. This involvement can for example be participation in political or trade union activities, sports clubs, or a religious organization, but it can also mean participation in local associations dedicated to managing or strengthening the neighbourhood area. It is a known fact that it is positive for one's health to have access to social support, and to be a part of social networks [45].

#### Self-rated health

*Self-rated health *is used as the dependent variable. It is a measure that comprises both physical and mental health [46] and predicts mortality [47].

#### Causal associations

There is an implicit assumption of a causal association in the conceptual model (the solid arrows). In several cases, however, causal associations go both ways, which is illustrated with the dotted arrows. A person with poor health, for instance, may be less likely to have an appropriate education, and to obtain well-paid employment or start a family. There may also be a reverse causality between health risk factors and self-rated health, but also between health risk factors and certain individual factors. It is for instance more difficult for people with poor health to be physically active, and alcohol problems can lead to problems within the family or at the workplace. However, in this cross-sectional study it is only possible to make statements about associations between different factors and self-rated health.

## Methods

### Study population

The association between self-rated health, individual factors, health risk factors, neighbourhood antecedent factors, social cohesion and social capital was investigated in a population sample of women and men aged 18-84 years in Örebro municipality, Sweden. The data were collected with a postal questionnaire from August to October 2004. The sampling procedure used was an independent random sample stratified by sex, age group, and geographical area. The data collection ended after two postal reminders. The individuals in the sample were informed through the covering letter that sex, age, geographical area, education level, occupation, and native country would be linked to their answers from the Swedish official registries if they responded to the questionnaire. The respondents thus accepted the linking of official registry data to the questionnaire data by informed consent. The personal identification numbers were deleted directly after the record linkage with the national registers and the survey data are thus anonymous. The survey was approved by the boards of the Uppsala County Council, Sörmland County Council, County Council of Västmanland, Värmland County Council and Örebro County Council. The strict Swedish law (Sekretesslagen 1980: 100 9 kap. 4§ [The law of secrecy]; Lagen om Officiell statistik 2001: 99 6§ [The law of official statistics]) assured that the study was conducted under strict ethical principles. Statistic Sweden, the statistical administrative authority in Sweden, carried out the sampling, collected the data and delivered de-identified data to the county councils. This was done under the jurisdiction of the Swedish law, the Helsinki declaration and international guidelines. An approval from an ethics committee was not applicable because the data are anonymous.

The study population consisted of the respondents who lived in one of four types of housing areas: City centre, Flats (mostly blocks of rental flats), Residential (mostly one-family private houses) and Outlying (suburbs and rural areas) in the municipality of Örebro, Sweden. This grouping of neighbourhoods was used in previous studies [28-30] and is based on the grouping that Örebro city council used to define the different housing areas. These areas are composed of small administrative geographical areas, key-codes, with similar characteristics but not necessary adjacent. This means that the areas include similar independent localities where individual observations in each locality are not independent.

The total response rate was 64.2% (Table 1). The total number of respondents was 3327 but only 2346 were included in the study population due to the fact that they answered all questions included in the analysis. The total response rate differed between women and men as well as housing areas. Moreover, it also varied between different age groups, for instance older people were more likely to respond to the questionnaire than younger people.

**Table 1:** Total number of respondents and response rates by housing area for women and men in the "Life and Health 2004" survey

	Total number of respondents			Response rate (%)		
	
Housing area	Women	Men	Total	Women	Men	Total
City centre	364	313	677	70.1	60.5	65.3
Residential	367	349	716	63.0	53.5	58.2
Flats	655	552	1207	71.0	67.1	69.0
Outlying	376	351	727	72.6	67.8	70.2

Total	1762	1565	3327	67.9	60.5	64.2

### Measures

The components in the conceptual model consist of several factors (Table 2). Registry data from Statistics Sweden regarding the respondents' native country have been used to create the factor *Country of birth*. If available, registry data from Statistics Sweden were used to create the factor *Education*. If not available, which is the case for some of the elderly 80-84 years of age, the self-reported highest education from the questionnaire was used. The factor *Housing area *has been constructed from information about which key-code area the respondent was living in as of August 2004. All other factors have been constructed from the self-reported answers to the survey questionnaire.

**Table 2:** Distribution of respondents by explanatory variables (n, %), and odds ratios (OR) with 95% confidence intervals (95% CI) of bivariate associations between explanatory variables and the outcome of poor self-rated health

			Respondents (N = 2346)			
			n	%	OR	(95% CI)
	Sex	Women (ref)	1259	53.7		
		Men	1087	46.3	1.1	(0.8-1.5)
	
	Age	18-34 (ref)	498	21.2		
		35-49	558	23.8	2.0 *	(1.1-3.6)
		50-64	632	26.9	3.0 ***	(1.7-5.3)
		65-79	540	23.0	2.2 **	(1.2-4.0)
		80-84	118	5.0	5.1 ***	(2.5-10.3)
	
	Education	High (ref)	761	32.4		
		Medium	971	41.4	2.1 **	(1.3-3.3)
**1.i **Individual factors - Socio-demographic factors		Low	614	26.2	3.2 ***	(2.0-5.1)
	
	Country of birth	Sweden (ref)	2114	90.1		
		Other Nordic countries	67	2.9	3.4 ***	(1.8-6.5)
		Outside the Nordic countries	165	7.0	2.3 **	(1.4-3.7)
	
	Family structure	Living with partner and children under 18 years (ref)	530	22.6		
		Single, not living with children under 18 years	518	22.1	2.9 ***	(1.8-4.9)
		Single, living with children under 18 years	132	5.6	3.1 **	(1.6-6.2)
		Living with partner and not with children under 18 years	1053	44.9	1.6	(1.0-2.7)
		Living with other adult	113	4.8	0.4	(0.1-1.9)
	
	Economic stress	No problem with current bills the last 12 months (ref)	1952	83.2		
		Problems 1-12 months	394	16.8	2.9 ***	(2.0-4.1)
	
	Employment status	Employee (ref)	1093	46.6		
		Unemployed	135	5.8	1.9	(0.9-4.2)
		Disability pensioner (retired)	121	5.2	20.6 ***	(12.5-33.8)
		Other	997	42.5	2.2 ***	(1.5-3.4)

	Critical life events	No critical life event (ref)	1025	43.7		
**1.ii **Individual Factors - Percieved life events		Critical life event	1321	56.3	1.6 **	(1.2-2.1)
	
	Accidents	No accident (ref)	2157	91.9		
		Been involved in accident	189	8.1	1.3	(0.7-2.2)
	
	Violence (assaults)	Not been exposed to violence (ref)	2304	98.2		
		Been exposed to violence	42	1.8	1.9	(0.7-4.8)
	
	Burdensome domestic work	Does not experience domestic work as burdensome (ref)	1202	51.2		
		Experiences domestic work as burdensome	1144	48.8	2.1 ***	(1.5-2.9)

	Alcohol habits (AUDIT)	No risky alcohol habits(ref)	2202	93.9		
		Risky alcohol habits	144	6.1	0.9	(0.4-1.8)
	
	Smoking habits	Not daily smoker (ref)	1997	85.1		
		Daily smoker	349	14.9	1.9 **	(1.3-2.7)
**2 **Health behaviour						
	
	Physical activity	Physically active (ref)	1958	83.5		
		Physically inactive	388	16.5	2.7 ***	(1.9-3.8)
	
	Body Mass Index	Normal weight (ref)	1201	51.2		
		Underweight	25	1.1	3.2 *	(1.1-9.5)
		Overweight	831	35.4	1.3	(0.9-1.9)
		Obesity	289	12.3	1.8 *	(1.1-2.8)

	Housing area	City centre (ref)	519	22.1		
**3.i **Neighbour hood anteceden t factors - Structural factors		Residential	532	22.7	1.0	(0.6-1.9)
		Flats	779	33.2	2.8 ***	(1.7-4.5)
		Outlying	516	22.0	1.8 **	(1.1-3.2)
	
	Home ownership	Own the dwelling (ref)	1046	44.6		
						
		Rent the dwelling	1300	55.4	2.0 ***	(1.4-2.9)
	
	Residential stability	6 years or longer (ref)	1377	58.7		
		1-5 years	725	30.9	1.3	(0.9-1.8)
		Shorter than 1 year	244	10.4	0.8	(0.4-1.5)

	Dwelling satisfaction	Enjoy present dwelling (ref)	2134	91.0		
**3.ii **Neighbour hood anteceden t factors - Percieved features		Do not enjoy present dwelling	212	9.0	1.9 **	(1.2-2.9)
	
	Satisfaction with dwelling surroundings	Enjoy present dwelling surroundings (ref)	2092	89.2		
		Do not enjoy present dwelling surroundings	254	10.8	2.4 ***	(1.6-3.6)
	
	Environmental problems in dwellings	No problems (ref)	2029	86.5		
		1-3 problems	317	13.5	1.9 **	(1.3-2.8)
	
	Environmental problems in dwelling surroundings	No problems (ref)	1187	50.6		
		1-3 problems	1159	49.4	1.5 *	(1.1-2.1)
	
	Environmental problems in housing area	No problems (ref)	1621	69.1		
		1-3 problems	725	30.9	2.1 ***	(1.5-2.9)

	Trust	Good trust (ref)	1949	83.1		
		Not good trust	397	16.9	2.0 ***	(1.4-2.8)
**4 **Social coheision						
	
	Conectedness	Good conectedness (ref)	1231	52.5		
		Not good conectedness	1115	47.5	1.2	(0.9-1.6)
	
	Unafraid	Unafraid of going out (ref)	1824	77.7		
		Afraid	522	22.3	1.6 *	(1.1-2.2)

**5 **Social capital	Personal support	Has personal support (ref)	2222	93.2		
		Does not have personal support	161	6.8	4.6 ***	(2.9-7.2)
	
	Pride	Has been made proud (ref)	1882	80.2		
		Has not been made proud	464	19.8	2.7 ***	(1.9-3.8)
	
	Participation	Participates in associations (ref)	980	41.8		
		Does not participate	1366	58.2	1.9 **	(1.3-2.7)

Level of significance for OR: *(p < 0.05) **(p < 0.01) ***(p < 0.001)

For each factor the category with the lowest proportion of poor self-rated health was chosen as the reference group.

#### Individual factors

#### Socio-demographic factors

##### Age and sex

The respondents are divided into five age groups 18-34, 35-49, 50-64, 65-79 and 80-84, where the youngest age group is the reference group. Women are the reference group in relation to men.

##### Education

Compulsory school or equivalent education for nine years or less is included in the low education group. The medium educational level is equivalent to comprehensive school. The people with a higher educational level than comprehensive school are included in the high educational level (reference group).

##### Country of birth

The reference group is people born in Sweden. To avoid excessively small study groups, only two more categories were used; people born outside Sweden but in the Nordic countries, and people born outside the Nordic Countries.

##### Family structure

This factor is constructed from two questions. The first question concerns the person with whom the respondent is living and their relationship (marriage, parenthood etc.). The second asks whether the respondent is living, part or full time, with children, own or others, under the age of 18 years. From these two questions the respondents were divided into five groups. The reference group is people living with a partner and children under the age of 18 years.

##### Economic stress

A binary factor was constructed to measure economic stress with people having problems paying their bills for at least one of the previous 12 months as one group compared to a reference group of those who did not have any such problems.

##### Employment status

This factor consists of four groups, where employees and those who manage ones own business is the reference group, which is compared to disability pensioners, unemployed and others (students, pensioners, people on parental leave and those doing unpaid domestic work at home).

#### Perceived life events

##### Critical life events

The respondents who answered that during the past two years they had lost a next of kin, had a serious disease, had a relative with a serious disease, separated from a partner, been dismissed or lost their employment or experienced some other painful or critical event have been defined as having experienced a critical life event. People who had not experienced a critical life event during the past two years are the reference group.

##### Accidents

The respondents who during the past twelve months had an accident that forced them to seek medical or dental care are one group, and those who answered negatively to the question are the reference group.

##### Violence (assaults)

The respondents who during the past twelve months had been exposed to violence or assaults are one group and the respondents who answered negatively to the question are the reference group.

##### Burdensome domestic work

The respondents who experienced their domestic work as burdensome some, most or all of the time were aggregated into one group. The people who answered the question with seldom or never make up the reference group.

#### Health risk factors

##### Alcohol habits

Alcohol habits were estimated from the answers to the first three questions from the Alcohol Use Disorders Identification Test (AUDIT) [48]. An index is calculated from the questions about how often they drink, how much is consumed on a normal occasion and how often a larger amount of alcohol is consumed. The index varies from 0-12. Women who have 6-12 points and men who have 8-12 are categorized as high risk consumers. The others are the reference group. For the last decade it has been common in Swedish population surveys to estimate high-risk alcohol use in this way [49].

##### Smoking habits

This factor was dichotomized into daily smokers and others (reference group).

##### Physical activity

Respondents with low physical activity in their leisure time (walking, cycling, or other light exercise less than 2 hours a week) defined the study group and those with a higher level of exercise were the reference group.

##### Body mass index (BMI)

From the respondents´ self-reported weight and height, a body mass index, (BMI = kg/m^2^) was calculated. BMI was categorized according to the WHO guidelines as underweight when BMI was <18.5, normal weight when BMI was between 18.5 and 24.9 (reference group), overweight when BMI was between 25 and 29.9, and obesity when BMI was equal to or more than 30 [50].

#### Neighbourhood antecedent factors

#### Structural factors

##### Housing area

Areas with similar characteristics were defined by small administrative geographical areas, key-codes. The factor consists of the areas: City centre, Flats (mostly blocks of rented flats), Residential (mostly one-family private houses) and Outlying (suburbs and rural areas). The City centre area is the reference group.

##### Home ownership

The people who answered that they owned their home are the reference group in relation to those with rented homes.

##### Residential stability

Those who answered that they had been living in the same housing area for more than 5 years are the reference group and the other two groups are people who lived in the same housing area for 1-5 years and shorter than 1 year.

#### Perceived features

##### Dwelling satisfaction

The people who responded that they enjoy their present residence 'very much' or 'rather much' are the reference group compared to people who answered 'neither good or bad', 'rather bad' or 'very bad'.

##### Satisfaction with dwelling surroundings

People who enjoyed the surroundings around their present residence 'very much' or 'rather much' are the reference group as opposed to people who did not.

The three factors that concern different problems connected to the dwellings are all options to the following question: 'Do you experience some of the following disturbances in or around your residence?' All three factors have been dichotomized in the same manner. The reference group is defined as people who answered 'seldom' or 'never' to all three questions that were used to assess the factor.

##### Environmental problems in dwellings

This factor is assessed with three questions about problems with cold draughts and low temperatures in the residence, bad smell in the residence and distasteful drinking water.

##### Environmental problems in dwelling surroundings

The three questions concern problems with noise from outside, exhaust from outside and disturbing industry.

##### Environmental problems in housing area

This factor was estimated by three questions asking whether the respondent experienced problems with disturbing neighbours, litter in the surroundings and vandalism or graffiti.

#### Social cohesion

##### Trust

People who agreed with both the statements 'The people in this neighbourhood can be trusted' and 'In this neighbourhood you can feel safe and confident that you will not be assaulted or threatened' are the reference group for the factor social trust. Other combinations of answers define the other group for this dichotomous factor.

##### Connectedness

People who agreed with both the statements 'The people in this neighbourhood know each other well' and 'The people in this neighbourhood care for each other' are the reference group for the dichotomous factor social connectedness.

##### Unafraid

People who answered that they never refrained from going out due to fear of being attacked, robbed or assaulted in some other way are the reference group for the dichotomous factor unafraid of going out.

#### Social capital

##### Personal support

The factor personal support is defined by a question determining whether the respondents believe that they have anyone who can help them in case of a personal problem or crisis in life. The question is dichotomized into a reference group, answering 'Quite sure' or 'Probably', and the other group, answering 'Probably not' and 'No'.

##### Pride

People who answered that during the past three months they had experienced that someone had made them feel proud of themselves are the reference group in this dichotomous factor.

##### Participation

The respondents who answered that they had been participating in activities or attending meetings in a group of different types of organizations are defined as the reference group for the factor participation in relation to those who did not.

#### Self-rated health

The outcome variable self-rated health was measured with the question 'How do you rate your general health?' The options 'Very poor' (n = 10, 0.4%), 'Poor' (n = 151, 6.4%), 'Neither good nor poor' (n = 501, 21.4%), 'Good' (n = 1250, 53.3%) and 'Very good' (n = 434, 18.5%) were dichotomized. The first two response options were grouped to indicate poor self-rated health and the remaining options were grouped into one group.

### Analytical procedures

We have tried to reduce our analytical model as much as possible without losing any of the dimensions included in the conceptual model (Figure 1). To achieve sufficiently large groups for the analysis we have dichotomized several of the factors (Table 2).

The analysis has been conducted by multivariate logistic regression (Method: Forced entry) in seven model steps with self-rated health as the dependent variable. The first model was conducted with only the individual socio-demographic factors followed by a new dimension for each model step. The analytic order thus starts with individual factors and health risk factors that are strongly related to self-rated health. The next component that is introduced in the analysis, neighbourhood antecedent factors, consists of two dimensions. Finally the two components social cohesion and social capital are included in the analysis.

## Results

The crude odds ratios for all 29 factors and poor self-rated health have been calculated (Table 2) and several of the factors show significant values. Many of the factors which in the bivariate case have a statistically significant association to poor self-rated health are not significant in the following multivariate analysis. This is the case for the individual factor critical life event. Among the health risk factors, the groups that are significant only in the bivariate case are daily smokers, obese people and people with underweight. In the dimension neighbourhood structural factor, the group that rent their homes is significantly associated with poor health in the bivariate case but not in the multivariate analysis. In this dimension there is one group, to have lived in the same area for 1-5 years, which has an association with poor health only in the multivariate analysis.

All five factors included in the perceived features of the neighbourhood show significant crude odds ratios, but none remain significant in the final model. Two of the three factors included in social cohesion show significant crude odds ratios, trust and unafraid. None of the factors included in social cohesion have significant odds ratios in the multivariate analysis. The factor participation, included in social capital, has a significant odds ratio only in the bivariate case.

Guided by our conceptual model (Figure 1), the multivariate analysis has been conducted in several model steps. *Model 1i *shows the individual socio-demographic factors that have strong relations to self-rated health (Table 3). There are five factors: age, country of birth, family structure, economic stress and employment status that have groups which have significant odds ratios (OR) throughout the whole analytic procedure. The groups that remain significant in all model steps are: aged 80-84 years, born outside Sweden but in the Nordic countries, living alone, having economical problems and being a disability pensioner. In *model 1ii*, perceived life events, the factor burdensome domestic work is significant and remains significant throughout all model steps of the analysis (OR in final model = 2.2 with 95% CI 1.5-3.3).

**Table 3:** Multivariate logistic regression models: Odds ratios (OR) for poor self-rated health in each model, with 95% confidence intervals (95% CI) for the final model

			(1.i)	(1.i-1.ii)	(1i-2)	(1.i-3.i)	(1.i-3.ii)	(1.i-4)		(1.i-5)
			OR	OR	OR	OR	OR	OR	OR	(95%CI)
	
	Sex	Women (ref)								
		Men	1.3	1.5 *	1.5 *	1.5 *	1.5 *	1.5 *	1.4	(0.9-2.1)
	
	Age	18-34 (ref)								
		35-49	1.5	1.5	1.5	1.8	1.9	1.9	1.7	(0.8-3.6)
		50-64	1.8	1.9	1.8	2.3 *	2.4 *	2.4 *	2.0	(1.0-4.2)
		65-79	2.1	2.3 *	2.3 *	2.9 *	3.3 **	3.3 **	2.7 *	(1.1-6.6)
		80-84	4.2 **	4.5 **	4.2 **	5.6 **	6.8 ***	6.7 ***	5.6 ***	(2.0-15.7)
	
	Education	High (ref)								
		Medium	1.4	1.5	1.4	1.3	1.3	1.3	1.1	(0.7-1.9)
**1.i **Individual factors - Socio-demographic factors		Low	2.0 *	2.1 *	1.9 *	1.7	1.7	1.7	1.5	(0.8-2.6)
	
	Country of birth	Sweden (ref)								
		Other Nordic countries	2.3 *	2.6 *	2.7 **	2.6 *	2.4 *	2.5 *	2.5 *	(1.2-5.5)
		Outside the Nordic countries	2.3 **	2.2 **	2.1 *	1.8	1.7	1.6	1.4	(0.7-2.6)
	
	Family structure	Living with partner and children under 18 years (ref)								
		Single, not living with children under 18 years	2.1 *	2.5 **	2.5 **	2.4 *	2.5 *	2.5 *	2.3 *	(1.1-4.6)
		Single, living with children under 18 years	2.1	2.1	2.3 *	2.1	2.0	2.0	1.8	(0.8-4.1)
		Living with partner and not with children under 18 years	1.4	1.7	1.7	1.8	1.8	1.8	1.7	(0.9-3.4)
		Living with other adult	0.5	0.5	0.6	0.6	0.7	0.7	0.7	(0.2-3.2)
	
	Economc stress	No problem with current expenses the last 12 months (ref)								
		Problems 1-12 months	2.9 ***	2.7 ***	2.9 ***	2.7 ***	2.6 ***	2.6 ***	2.6 ***	(1.6-4.2)
	
	Employment status	Employee (ref)								
		Unemployed	1.3	12.2	1.1	1.1	1.3	1.0	1.0	(0.4-2.3)
		Disability pensioner (retired)	13.0 ***	11.2 ***	12.0 ***	11.2 ***	11.5 ***	11.5 ***	11.4 ***	(6.4-20.1)
		Other	1.4	1.4	1.4	1.4	1.4	1.3	1.3	(0.7-2.5)
	Critical life events	No critical life event (ref)								
		Critical life event		1.3	1.3	1.3	1.3	1.3	1.3	(0.9-1.9)
	
**1.ii **Individual Factors - Percieved life events	Accidents	No accident (ref)								
			Been involved in accident	1.2	1.1	1.2	1.2	1.1	1.3	(0.7-2.4)
	
	Violence (assaults)	Not been exposed to violence (ref)								
		Been exposed to violence		1.9	2.3	2.1	2.1	2.2	2.2	(0.7-7.0)
	
	Burdensome domestic work	Does not experience domestic work as burdensome (ref)								
		Experiences domestic work as burdensome		2.4 ***	2.3 ***	2.4 ***	2.3 ***	2.3 ***	2.2 ***	(1.5-3.3)

	Alcohol habits (AUDIT)	No risky alcohol habits(ref)								
		Risky alcohol habits			0.7	0.7	0.7	0.7	0.6	(0.3-1.5)
	
	Smoking habits	Not daily smoker (ref)								
		Daily smoker			1.0	1.0	1.0	0.9	1.0	(0.6-1.7)
	
**2 **Health behaviour	Physical activity	Physically active (ref)								
		Physically inactive			2.2 ***	2.2 ***	2.1 ***	2.2 ***	2.1 ***	(1.4-3.3)
	
	Body Mass Index	Normal weight (ref)								
		Underweight			2.3	2.3	2.1	2.1	1.9	(0.5-7.5)
		Overweight			1.0	1.0	0.9	0.9	1.0	(0.6-1.5)
		Obesity			0.9	0.9	0.8	0.8	0.9	(0.5-1.6)

	Housing area	City centre (ref)								
		Residential				2.0	2.1 *	2.0	1.9	(0.9-4.1)
**3.i **Neighbourhood antecedent factors - Structural factors		Flats				1.9 *	1.8 *	1.7	1.8 *	(1.0-3.2)
		Outlying				2.5 *	2.7 **	2.6 **	2.5 **	(1.3-5.1)
	
	Home ownership	Own the dwelling (ref)								
		rent the dwelling				1.6	1.4	1.5	1.4	(0.8-2.6)
	
	Residential stability	6 years or longer (ref)								
		1-5 years				1.7 *	1.7 *	1.7 *	1.7 *	(1.1-2.6)
		Shorter than 1 year				1.1	1.1	1.1	1.1	(0.5-2.4)

	Dwelling satisfaction	Enjoy present dwelling (ref)								
		Do not enjoy present dwelling					0.9	0.9	0.8	(0.4-1.5)
	
**3.ii **Neighbourhood antecedent factors - Percieved features	Satisfaction with dwelling surroundings	Enjoy present dwelling surroundings (ref)								
	
		Do not enjoy present dwelling surroundings					1.4	1.4	1.4	(0.7-2.5)
	
	Environmental problems in dwellings	No problems (ref)								
		1-3 problems					1.3	1.3	1.4	(0.8-2.3)
	
	Environmental problems in dwelling surroundings	No problems (ref)								
		1-3 problems					1.2	1.2	1.2	(0.8-1.8)
	
	Environmental problems in housing area	No problems (ref)								
		1-3 problems					1.4	1.4	1.4	(0.9-2.1)

	Trust	Good trust (ref)								
		Not good trust						1.2	1.3	(0.8-2.1)
**4 **Social coheision	Conectedness	Good conectedness (ref)								
		Not good conectedness						0.8	0.7	(0.5-1.1)
	
	Unafraid	Unafraid of going out (ref)								
		Afraid						1.1	1.0	(0.7-1.6)

	Personal support	Has personal support (ref)								
		Does not have personal support							2.2 **	(1.3-3.9)
	
**5 **Social capital	Pride	Has been made proud (ref)								
		Has not been made proud							2.3 ***	(1.5-3.4)
	
	Participation	Participates in associations (ref)								
		Does not participate							1.2	(0.8-1.8)

Level of significance for OR: *(p < 0.05) **(p < 0.01) ***(p < 0.001)

Among the health behaviours that are introduced in *model 2*, 'physically inactive' is the only significant group (OR in final model = 2.1 with 95% CI 1.4-3.3).

In *model 3i *the structural factors related to the neighbourhood are introduced into the analysis. Two significant factors in this dimension are housing area and residential stability. People who live in the flats areas (OR in final model = 1.8 with 95% CI 1.02-3.2), outlying areas (OR in final model = 2.5 with 95% CI 1.3-5.1) and/or with residential stability 1-5 years (OR in final model = 1.7 with 95% CI 1.1-2.6) have a significant association to poor self-rated health.

None of the factors in *model 3ii*, perceived neighbourhood factors, and *model 4*, social cohesion are significant in any model step.

Two of the groups in the final *model 5*, where the component social capital is introduced, are statistically significant, lack of personal support (OR = 2.2 with 95% CI 1.3-3.9) and not having experienced that someone has made them feel proud (OR = 2.3 with 95% CI 1.5-3.4).

There are several other factors in the final model with significant OR (Hosmer and Lemeshow Test: X^2 ^= 6.30; Sign 8_df _= 0.61; Nagelkerke R^2 ^= 0.32). Many of them can be attributable to the individual socio-demographic factors such as employment status and age. Other groups with significant OR in this dimension are: having been born in a Nordic country outside Sweden, having economic problems and living alone. Experiencing domestic work as burdensome in the dimension perceived life events and, being physically inactive in the component health risk factors, are also significant in the final model. Among the structural factors in the neighbourhood two are significant in the final model: housing area, with the groups who live in areas with predominately flats or in outlying areas, and residential stability, with the group 1-5 years.

## Discussion

Health is unequally distributed in the population and studies have shown that health covaries with neighbourhood factors [43,44]. The point of departure in this study is a conceptual model inspired by the work of Carpiano [31,32], in which different factors related to the neighbourhood have been used to find associations to individual self-rated health. An important point of view in this analysis is the availability - at an individual level - of social capital both connected to the peoples' neighbourhood and as personal support without any necessary connection to the living environment. Despite the fact that we have not used any multilevel data in this analysis, as in the original model, it has been a good starting point for analysing the relations between antecedent neighbourhood factors, social cohesion, social capital, individual factors, health risk factors and health.

### Factors that covary with health

The strongest factors connected to poor self-rated health in this study are found in the dimension of individual socio-demographic factors, which is in line with previous research [35]. Not surprisingly, the strongest results are found among disability pensioners and the elderly. To have some kind of illness is a precondition for being a disability pensioner, and high age is often connected with increased illness. Other strong individual factors are having been born in a Nordic country other than Sweden, living alone and having difficulties paying one's bills during the past year. It has previously been shown that being an immigrant and living under economic stress co-varies with poor health [30,33,51,52].

Despite the fact that we have very strong relations between explanatory (age and disability pensioners) and co-varying (immigrants and economically stressed) factors and poor self-rated health there are still some significant factors relating to housing area, residential stability and social capital. Poor health is more common among people living in areas with predominately large blocks of flats or areas outside the city. This is the only 'objective' measure connected to the neighbourhood area, since the other measures are based on self-reported information. It is also a measure which, after controlling for individual factors, can be interpreted as a contextual condition, meaning that it is something that exists independent of the people who live in the area. In the dimension of perceived features in the neighbourhood all factors were significant in the bivariate analysis but none remained in the final model. Despite this, according to the results from the crude odds ratios, taking actions regarding problems in the housing area and in its surroundings could be seen as a way to create a better environment for people living there [53,54].

Factors included in the component social cohesion covary with poor health only in the bivariate analysis. It is more common to report poor health among people who have a low sense of social trust in the neighbourhood or who answered that they sometimes are afraid of going out, indicating low confidence in the people in the neighbourhood. A plausible explanation as to why social cohesion does not remain significant in the final model is that there are several other factors in the model with a stronger association to health. The association between social cohesion and self-reported health seems to be indirect and mediated by other factors in the final model. However, in the component social capital both personal support and pride, in this case lack of support and to not have been made proud, are associated with poor self-rated health even after considering all other factors. This shows once again the importance of social support regarding individual health. To promote environments in the neighbourhood where social encounters can take place would be an approach to promoting pro-social and sustainable neighbourhoods [55,56].

### Strengths and limitations of the study

There is an implicit assumption that the neighbourhood area and the individual access to social capital can influence health in our conceptual model. However, the results are based on cross-sectional data, which limits the possibility of drawing causal conclusions. What we can study are the associations between health and factors relating to neighbourhood characteristics and social capital by controlling for a number of individual factors which are known to covary with health. A person with bad health can, for instance, find it difficult to move, even though it might be necessary to find more suitable housing. The associations may be spurious, potentially reverse casual or causal but our cross-sectional data can not be used to determine which.

One limitation is the response rate, which was 64.2%. The highest proportion of non-respondents is found among men in areas with predominately large blocks of flats and the lowest among women in the outlying areas. Women had a higher response rate than men within all the different housing areas. Moreover, it was more common among men to report poor health than among women. This is the opposite of the results of another study conducted in the middle of Sweden, which shows higher incidence of poor self-rated health among women than men [33]. There are also differences in response rates between different age-groups since older people are more prone to answer than younger people. This is a fact that implies that there may be a higher proportion of people with poor health in the population of Örebro. However, a high non-response rate does not necessarily imply response-bias. A meta-analysis of surveys did not find any clear association between response-rate and response-bias [57]. Analysis of associations seems in several studies be unaffected of low response-rates [58,59]. We have no reason to believe that this should not be the case in our study. Another limitation in our study is, due to the small number of geographical areas, that we could not conduct a multi-level analysis which would have required a larger sample size. We have also been unable to control for neighbourhood socioeconomic status and this may have contributed to confounding bias in the results.

One strength of this study is that our model seems to be relatively stable since several of the significant factors in the final model were significant from the first model in which they were introduced. The fact that the grouping of neighbourhoods, i.e. housing-areas, is based on objective measures is another strength. In the present study, data collected at the individual level are used to account for individual differences in the experience of neighbourhood aspects. It confirms the previously reported relationships between experiences of housing area and self-rated health [28-30]. Moreover, the results are also in line with other individual level analyses of social capital and self-reported health [60,61].

However, there are limits in both quantitative single and multilevel analysis more generally. The challenge is to understand the deep, complex, and dynamic interrelation between people and context. One concern relates to operationalising context in term of the hierarchical structure of the data [5], which in this study was limited to the four types of housing areas, where two remained significant in the final multivariate model. Another interpretation of this result could be that the housing-area effect, which remained after controlling for residential stability and home ownership, may be an artefact of some other unmeasured factors.

Moreover, the partial non-independence of observations may be another less likely explanation. A proper analysis of both the within and between-context variations require many individuals from many places [5]. In this study the number of housing-areas was too small for such analysis. The present analysis is based on a random sample of the population in the municipality and not a cluster sample from different housing areas. Another step towards analysis of the dynamic relationship between people and context is to develop sound observational measures of neighbourhood factors [62]. Such research will also need qualitative and other social science approaches [63,64].

In this study we have used sex as one underlying factor among others and found that it was not a significant factor in relation to poor self-rated health in the final model. In further studies we would like to analyse the material separately for women and men to determine if there are gender-specific factors that are important in the relations between neighbourhood antecedent factors, social cohesion, social capital and health. In this case it would not only be of interest to study self-rated health but also mental health since there are more pronounced differences in the mental health of women and men. Moreover, data collected in the third wave of the surveys of 'Life & Health' will give us opportunities to study if the relations we have found are stable or whether they are changing over time.

## Conclusions

Two of the three indicators of social capital, i.e. lack of personal support and no experience of being made proud, were related to poor health in the multivariate model, but this was not the case for any of the measures of social cohesion. The perceived neighbourhood features such as satisfaction with dwelling and its surroundings or perceived problems did not contribute to the final model in contrast to housing area and residential stability, which both made independent contributions to the final model for social capital processes on individual health. Moreover, designing and building healthy places is not a new concept as for centuries, those who care about health, across many professions, have turned their attention to the built environment. Communities can construct physical environments that promote social interaction and participation, and these in turn support the development of social networks, social support, sense of community, community competence and a sense of place, all of which are important determinants of health. It is possible that the distinction between 'composition' and 'context' may be more apparent then real, and that features of both material infrastructure and collective social functioning influence health. Whatever the reasons for area differences, it is clear that areas where vulnerable populations tend to live must be prioritized. Consequently, the neighbourhoods can be an important setting for health promotion as for many people, these are where social encounters take place.

## Competing interests

The authors declare that they have no competing interests.

## Authors' contributions

ML-B, CP and CE participated in the design of the study, acquisition of theoretical framework and survey data, statistical analysis and drafted the manuscript. All authors read and approved the final manuscript.

## Pre-publication history

The pre-publication history for this paper can be accessed here:

http://www.biomedcentral.com/1471-2458/10/628/prepub
